# Global pattern of phylogenetic species composition of shark and its conservation priority

**DOI:** 10.1002/ece3.1724

**Published:** 2015-09-23

**Authors:** Hungyen Chen, Hirohisa Kishino

**Affiliations:** ^1^ National Research Institute of Fisheries ScienceFisheries Research AgencyKanagawa236‐8648Japan; ^2^ Graduate School of Agricultural and Life SciencesThe University of TokyoTokyo113‐8657Japan

**Keywords:** Biodiversity, biogeography, community ecology, ecophylogenetic diversity, phylogenetic skew, shark conservation

## Abstract

The diversity of marine communities is in striking contrast with the diversity of terrestrial communities. In all oceans, species richness is low in tropical areas and high at latitudes between 20 and 40°. While species richness is a primary metric used in conservation and management strategies, it is important to take into account the complex phylogenetic patterns of species compositions within communities. We measured the phylogenetic skew and diversity of shark communities throughout the world. We found that shark communities in tropical seas were highly phylogenetically skewed, whereas temperate sea communities had phylogenetically diversified species compositions. Interestingly, although geographically distant from one another, tropical sea communities were all highly skewed toward requiem sharks (Carcharhinidae), hammerhead sharks (Sphyrnidae), and whale sharks (*Rhincodon typus*). Worldwide, the greatest phylogenetic evenness in terms of clades was found in the North Sea and coastal regions of countries in temperate zones, such as the United Kingdom, Ireland, southern Australia, and Chile. This study is the first to examine patterns of phylogenetic diversity of shark communities on a global scale. Our findings suggest that when establishing conservation activities, it is important to take full account of phylogenetic patterns of species composition and not solely use species richness as a target. Protecting areas of high phylogenetic diversity in sharks, which were identified in this study, could form a broader strategy for protecting other threatened marine species.

## Introduction

Global studies of predator diversity reveal predictable patterns, which suggest that there will be ecosystem‐wide changes in response to changes in climate and fishing pressure. Worm et al. (2005) studied worldwide patterns of predator diversity (tuna and billfish) and revealed distinct subtropical hotspots, which appeared to hold generally for other predators. The diversity of tuna and billfish consistently peaked at intermediate latitudes (10–35°), which is similar to other pelagic taxa (Boyce et al. 2008). Trebilco et al. (2011) examined the interrelationships among species richness distributions of tuna and billfish species, fishing pressure, and increases in sea surface temperatures (SSTs) in tropical to temperate oceans. They found that in the Indian and Pacific Oceans, higher fishing pressure is associated with higher species richness. In the Pacific and Atlantic Oceans, species richness is generally higher in areas that have seen lower levels of change in SST. In addition, these investigations provided evidence that ambient water temperature tolerances of tuna and billfish can be used to predict broad species richness patterns on a global scale.

The primary index used to measure community diversity is species richness, which is the number of member species. Tittensor et al. (2010) studied species richness of 13 major species groups, ranging from zooplankton to marine mammals. They found that coastal species showed maximum diversity in the Western Pacific, whereas oceanic groups consistently peaked across broad midlatitudinal bands in all oceans. To protect the full range of biodiversity, conservation strategies cannot be based solely on hotspots of species richness, but must also consider other biodiversity hotspots (Kareiva and Marvier 2003), as well as endemism and human impacts (Trebilco et al. 2011; Selig et al. 2014). In addition to monitoring species richness, comprehensive conservation activities should monitor species compositions carefully.

Sharks play a crucial role in maintaining the health of marine environments. However, one‐third of all shark species are threatened or near‐threatened because of commercial and recreational fishing. Shark and ray landings are steadily increasing from 1950 to the peak year in 2003 and subsequently declined 15% by 2011 (Davidson et al. 2015). Chondrichthyan fishes (sharks, rays, and chimeras) extinction risk is substantially higher than most other vertebrates, and only one‐third of species are considered safe (Dulvy et al. 2014). Lucifora et al. (2011) identified hotspots of shark species richness, functional diversity, and endemicity, which may be regarded as priority areas for conservation. Shark species richness is highest on the continental shelves and at intermediate latitudes (Lucifora et al. 2011), which is uniquely different from terrestrial communities (Rohde 1992) and other marine taxa (Tittensor et al. 2010; Selig et al. 2014), which are most diverse in the tropics. Sharks are the most diverse group of large predatory animals and play an important ecological role as the primary predators of many species (Myers and Worm 2003; Myers et al. 2007). Furthermore, the development of conservation strategies relies on knowledge of species evolutionary history and the status of their close relatives (Vélez‐Zuazo and Agnarsson 2011). Therefore, information on population genetic structure and connectivity should be considered when establishing the conservation priorities of sharks.

Since the first proposal that phylogenetic diversity can serve as an additional component for nature conservation (Vane‐Wright et al. 1991), research efforts on the applicability of phylogenetic diversity to various ecological issues has steadily increased (Bininda‐Emonds et al. 2000; Winter et al. 2013). As phylogenies reflect integrated phenotypic differences among taxa, evolutionary relationships may be related to ecological processes and dynamics (Felsenstein 1985; Harvey and Pagel 1991; Faith 1992). Among its other applications, phylogenetic diversity can be used to address questions related to community large‐scale spatial patterns and be referred when establishing conservation activities (Knapp et al. 2008; Crisp et al. 2009; Morlon et al. 2011; Brum et al. 2012).

Faith's phylogenetic diversity (PD; Faith 1992) and average taxonomic distinctiveness (AvTD; Pienkowski et al. 1998) are the two most widely used phylogenetic diversity indices. PD, which measures community phylogenetic richness, is calculated as the sum of the lengths of all those branches that are members of the corresponding minimum spanning path (Faith 1992). Compared with species richness alone, using PD leads to the selection of different conservation priorities and greater preservation of feature diversity (Forest et al. 2007). AvTD measures community phylogenetic distinctiveness; it is calculated as the sum of all branch lengths connecting two randomly chosen species averaged across all species representing the mean distance between those two species. PD is mathematically related to species richness (Schweiger et al. 2008). In contrast, AvTD is independent of species richness; however, the extinction of closely related species will increase the index value. Furthermore, AvTD is related to trophic diversity (Pienkowski et al. 1998).

Phylogenetic skew (PS; Chen et al. 2015) is a measure of the species composition of a community, which takes into account the species composition of a set of target communities, known as meta‐communities. It compares the distribution of divergence times with the expected distribution, which assumes that the species composition of a community is obtained by random sampling from the meta‐community. If the member species of a community are aggregated in the meta‐community tree, on average, the divergence times between the member species are young. Conversely, if they are dispersed over multiple phylogenetic clusters, the divergence times of the member species tend to be old. Therefore, a large PS value is likely to represent a species composition that is phylogenetically skewed. In a comparative study, we also used the inverse of PS, which we call phylogenetic‐clade evenness (PE). The terms of phylogenetic evenness were used to describe the abundance‐based distribution of species in a community (Webb and Pitman 2002; Cadotte et al. 2010). Phylogenetic‐abundance evenness (PAE) was proposed to describe phylogenetic evenness of the abundance distribution scaled by branch lengths, which evaluates the relationship between the abundance and the distribution of terminal branch lengths (Cadotte et al. 2010).

In this study, we applied phylogenetic diversity to identify worldwide shark species compositions. We analysed the global patterns of Faith's phylogenetic diversity, average taxonomic distinctiveness, phylogenetic skew and our proposed index, phylogenetic‐clade evenness, of sharks globally. We mapped the areas with the highest values of phylogenetic diversity indices. As a result, we determined global phylogenetic diversity hotspots for sharks. Combining the hotspots of phylogenetic diversity with the information of species richness, we suggest priorities for phylogenetic conservation for shark.

## Materials and Methods

### Species richness distribution and mitochondrial COI sequences of shark

Our study was inspired by Lucifora et al. (2009), who built the Shark Distribution Database, which represents the cumulative distribution of all known sharks. The database contains the geographic distribution of all 507 shark species known to date, compiled from searches of the scientific literature published since 1878 on the distribution ranges of the species.

We then obtained nucleotide sequences of the mitochondrial COI region for 236 of the 507 shark species from the National Center for Biotechnology Information (NCBI 2014) database (Data S1). For each species in the database, a grid of 1 × 1 latitude–longitude cells was superimposed on each species' distribution map, and a single data point was recorded in each cell where the species was present.

**Figure 1:**
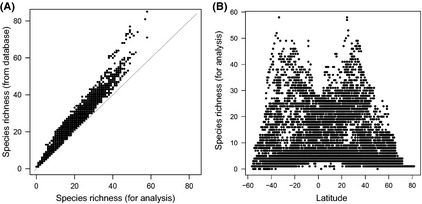
Relationship of the number of species used in our analysis (mitochondrial DNA COI sequences available species) with (A) the number of species in the global shark database and (B) latitude. Negative numbers indicate southern latitudes.

The number of species per cell, based on the Global Shark Distribution Database, ranged from 1 to 85 (Fig. S1A), while the number of analysed species (i.e., those with COI sequences) ranged from 1 to 58 per cell (Fig. S1B). Figure 1A shows the relationship between the number of species from the database and the number of analysed species (*r* = 0.99). In our analysis, shark species richness was highest at intermediate latitudes (Fig. 1B), which corroborates published species richness patterns from the database (Fig. 2A of Lucifora et al. 2011). We calculated the indices of phylogenetic diversity in each of the 5048 1 × 1 latitude–longitude grid cells with more than 10 species (Fig. S1C shows the number of analysed species ranked by percentiles).

**Figure 2:**
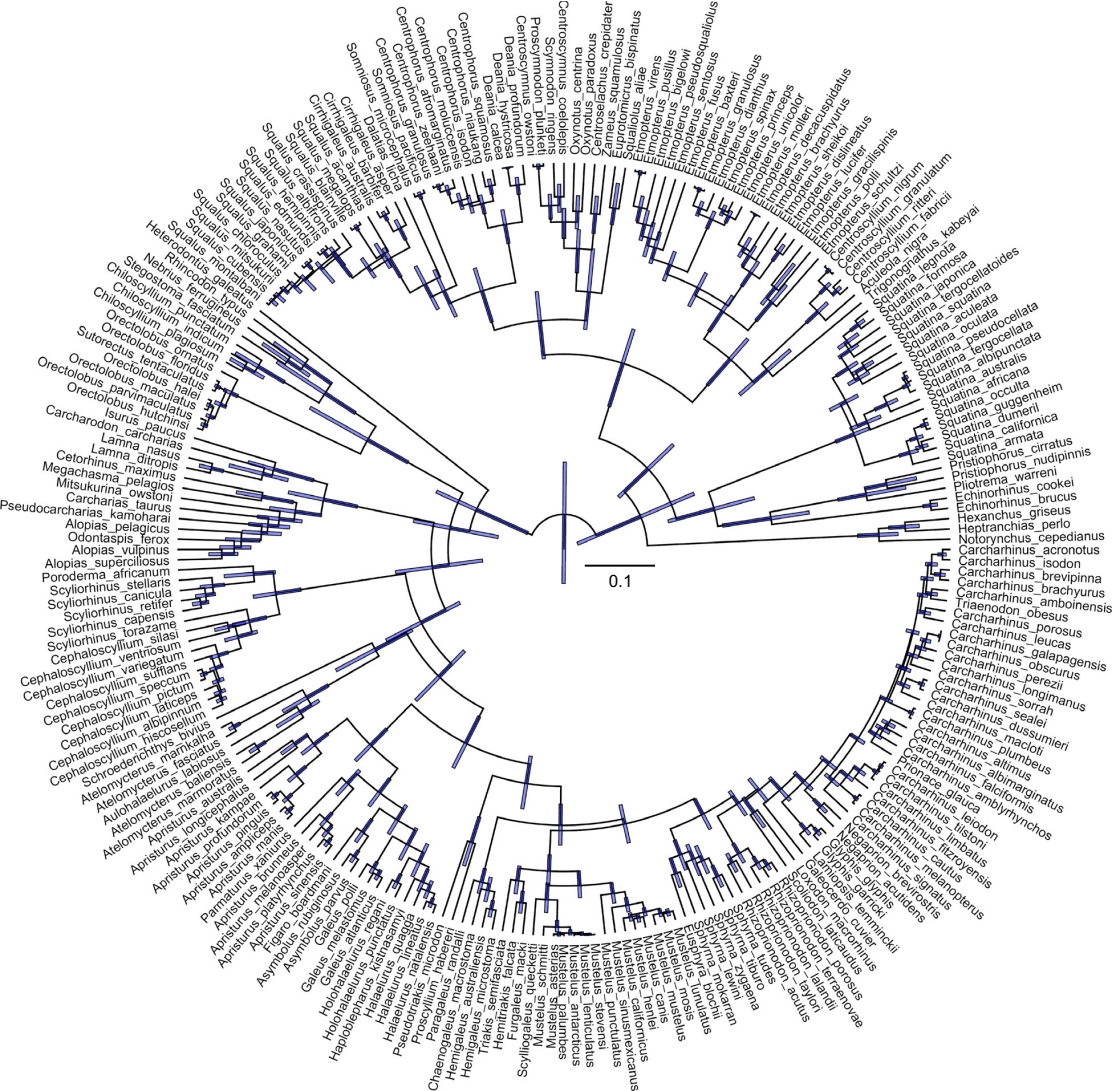
Bayesian phylogenetic tree of 236 shark species estimated from mitochondrial DNA COI sequences. The unit of lengths of the branches is the number of substitution per site. The bar represents the height of the 95% highest posterior density (HPD).

### Inference of phylogenetic tree and phylogenetic diversity indices

After performing sequence alignments using the MUSCLE program (Edgar 2004), implemented in MEGA 5.0 (Tamura et al. 2011), divergence times among shark species were estimated in a Bayesian framework using BEAST v1.7.5 (Drummond et al. 2012). The analysis used the HKY model of nucleotide substitution with gamma‐distributed rate heterogeneity among sites (Felsenstein 1981; Hasegawa et al. 1985; Yang 1994). A random local clock model was used to take account of variable evolutionary rates among lineages (Douzery et al. 2002; Drummond and Suchard 2010). The Yule process was used as the prior of the tree. As for the prior distributions of the parameters that specify the substitution process, we adopted the default values. The prior for the HKY transition‐transversion parameter was set to log‐normal distribution with a location parameter = 1 and a scale parameter = 1.25. The prior distribution of the shape parameter describing the heterogeneity rate among sites was the exponential distribution with mean = 0.5. The frequency of change in evolutionary rate followed a Poisson distribution with mean = 0.7. As we only use the relative values of the divergence times in the subsequent step, we set the mean evolutionary rate to 1. The Markov chain Monte Carlo (MCMC) chain length was set to 10,000,000. The topology was fully resolved without any polytomy.

Only cells with more than 10 species were used for building the maps, because of the small‐sample bias in the PS (Chen et al. 2015). The PD was calculated as the sum of the lengths of all branches that belonged to a corresponding minimum spanning path, which connected all the species recorded in each cell. AvTD was calculated for each cell as the sum of all branch lengths that connected two randomly chosen species, averaged across all species.

### Calculations of the phylogenetic skew and phylogenetic‐clade evenness using the Bayesian phylogenetic tree

The likelihood of the ordered divergence times of the sample by **t** = (*t*_1_, …, *t*_*s‐*1_), *t*_1_ > … > *t*_*s‐*1_, given speciation rate, *λ*, extinction rate, *μ*, species sampling proportion, *ρ*, and *t*_1_ using formulae of the generalized birth and death processes is obtained as (Eq. (1) in Chen et al. 2015; Kendall 1949; Nee et al. 1994; Yang and Rannala 1997),

(1) $$L_{0}\left(t|\lambda ,\mu ,\rho \right)=\left(s-2\right)!\prod_{j=2}^{s-1}\frac{\lambda p_{1}\left(t_{j}\right)}{v_{t_{1}}},$$

where

$$p_{1}\left(t\right)=\frac{1}{\rho }P{\left(0,t\right)}^{2}e^{(\mu -\lambda )t},$$

$$P\left(0,t\right)=\frac{\rho (\lambda -\mu )}{\rho \lambda +\left(\lambda \left(1-\rho \right)-\mu \right)e^{(\mu -\lambda )t}},$$

and

$$v_{t}=1-\frac{1}{\rho }P\left(0,t\right)e^{(\mu -\lambda )t}.$$

To calculate the PS and PE, we adopted the following two‐step procedure. In the first step, given the maximum likelihood estimates of speciation rate, $\hat{\lambda }$, and extinction rate, $\hat{\mu }$, the estimated effective species sampling proportion, ${\hat{\rho }}_{E}$, for each cell can be obtained by maximizing the Equation (1) using their distribution of divergence times. *ρ*_*E*_ explains differences in the distribution of divergence times of the community and that of the meta‐community (the species composition of a set of target communities) assuming that the species composition of the community is a random sample from the meta‐community. With *ρ* being fixed to 1, maximum likelihood estimates of speciation and extinction rates were $\hat{\lambda }$ = 17.08 ± 2.15 and $\hat{\mu }$ = 6.28 ± 3.51 (±SE), respectively. In the second step, we treated these estimates as fixed, and obtained the maximum likelihood estimate of ${\hat{\rho }}_{E}$ for each of the cells by maximizing Equation (1). The actual species sampling proportion, *ρ*_0_, was obtained by the ratio of the number of member species from the cell to the total number of species, 236. Therefore, we calculated PS = ${\hat{\rho }}_{E}/\rho_{0}$ and PE = $\rho_{0}/{\hat{\rho }}_{E}$ for each cell. All calculations were performed using the R program. To make all the indices comparable on the maps, each 1 × 1 latitude–longitude grid cell was colored to indicate rank of the value, in terms of percentiles.

## Results

### High phylogenetic skew of sharks in tropical seas

**Figure 3:**
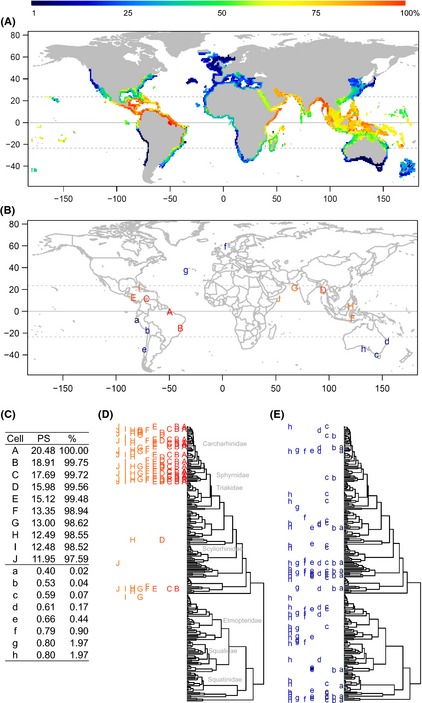
Global patterns of shark phylogenetic skew (PS). (A) Shark PS. (B) Map showing distributions of cells with high or low PS. (C) Values and percentile rank (%) of PS of cells shown in (B), (D), and (E). Species compositions of cells with high (D) and low (E) PS. Family names of major taxonomic groups are indicated the in the branches. The trees show phylogenetic relationships of 236 shark species derived from Bayesian analysis of mitochondrial DNA COI sequences (see Figure 1). Each 1 × 1 latitude–longitude cell is colored according to the percentile rank of PS. The letter is just used to identify different areas and the color represents the percentile rank of PS. The red cells represent the highest rank, while the blue color had the lowest rank.

We compiled COI sequences from 236 shark species, and constructed a Bayesian‐based phylogenetic tree of sharks worldwide (Fig. 2). We found that the PS of sharks was high along tropical coasts and low along temperate ones (Fig. 3A). The PS of sharks worldwide ranged from 0.40 to 20.48. Globally, the highest PS of sharks was found in the region of northern Brazil. High PS was also observed in other tropical locations, such as marine areas near eastern Brazil, Venezuela, Suriname, western India, Honduras, Indonesia, and Somalia. The lowest PS of sharks was found in temperate areas, including the North Sea and coastal regions of the United Kingdom, Ireland, southern Australia, and Chile.

To obtain further insights into shark PS, we examined the species compositions of cells with the highest and lowest PS values. Figure 3B shows the global distribution of the 10 (areas with PS > 97.5%) and eight (areas with PS < 2.5%) geographically distant areas with highest and lowest PS separately. A geographical distinction between regions with high and low PS was clearly evident: red cells (high PS) were located in the tropics and blue cells (low PS) were mainly located in temperate zones (the order of the letters is meaningless). Values and percentile rank of the cells are shown in Figure 3C.

Figure 3D,E show the species compositions of cells with high and low PS on the phylogenetic tree of 236 shark species separately. The letters are just used to identify different geographical areas and their colors represent the percentile rank of PS. We found that areas with high PS have similar species compositions, although the cells were situated in geographically separate, tropical locations. Orange and red letters (high PS, Fig. 3D) are clustered together mostly among Carcharhinoid and Sphyrnidae branches, and that blue letters (low PS, Fig. 3E) are more homogeneously distributed among all branches of the tree. That means, species compositions were highly assembled toward requiem sharks (Carcharhinidae), hammerhead sharks (Sphyrnidae), and whale shark (*Rhincodon typus*) in the areas with high PS. On the other hand, species in the cells with low PS were widely distributed across the phylogenetic tree, a pattern closely resembling that obtained by random or systematic species sampling.

### Global patterns of shark phylogenetic diversity indices

**Figure 4:**
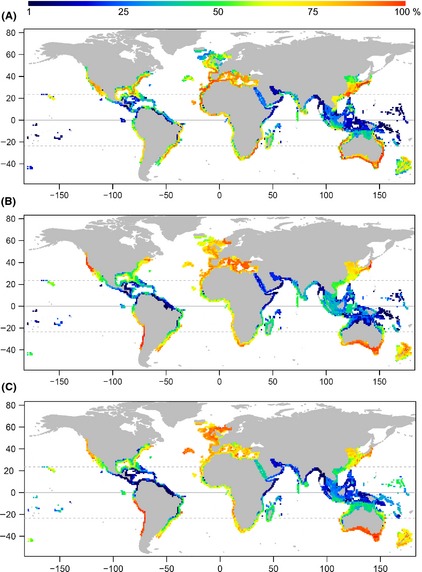
Global patterns of shark phylogenetic diversities. (A) Faith's phylogenetic diversity (PD). (B) Average taxonomic distinctiveness (AvTD). (C) Phylogenetic‐clade evenness (PE). Each 1 × 1 latitude–longitude cell is colored as the percentile rank of the index. The red cells represent the highest rank, while the blue color had the lowest rank.

Faith's PD was high near southern Japan, Taiwan, southern Australia, northwestern and southeastern Africa, and eastern United States, with the greatest value obtained off the coast of eastern Australia (Fig. 4A). The AvTD was high off the coasts of subtropical and temperate regions such as Chile, western United States, southern Australia, Argentina, Libya, southern Italy, eastern Canada, and southern Norway. The greatest value was obtained along the coast of eastern Japan (Fig. 4B). High PE was observed in temperate areas, such as the North Sea and coastal regions of the United Kingdom, Ireland, southern Australia, and Chile (Fig. 4C). Globally, these areas showed the greatest PE of sharks.

### Areas of high diversities from multiple perspectives

To identify areas of high priority for shark conservation, we first identified those cells with the highest (95th percentile) species richness (Fig. S2A). We selected a 5% threshold because it has been previously used in terrestrial (Jenkins et al. 2013), marine (Selig et al. 2014), and shark (Lucifora et al. 2011) analyses. This threshold was specific enough to ensure that we could separate very high diversity areas, but broad enough to enable the identification of multiple areas in different regions. We then identified those cells with the highest PD (95th percentile, top 203), to derive a structured framework for area prioritization with the greatest phylogenetic diversity (Fig. S2B). Next, to include information on species composition in the area prioritization, we identified those cells with the highest AvTD and PE separately (95th percentile, top 203, Figs S2C,D). High AvTD means high taxonomic distinctiveness in a sample, which represents phylogenetic endemism and high rarity in the area. However, high PE represents a balanced phylogenetic composition of species in a sample.

**Figure 5:**
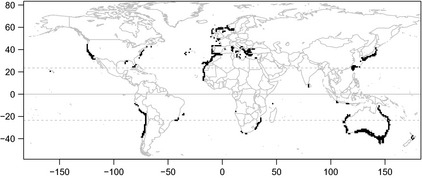
Phylogenetically informed priority areas for shark conservation. Each cell was selected because it contained the values in the top 5% of phylogenetic diversity (PD), average taxonomic distinctiveness (AvTD), phylogenetic‐clade evenness (PE), or species richness. See Figure S2 for maps of the top 5% of each index.

Based on these criteria, we identified 477 phylogenetic priority cells (11.2% of the cells with > 10 species) with the highest PD, AvTD, or PE. To include information on species richness in the inspection, and thus combining both hotspots of phylogenetic diversity and species richness, we identified 533 priority cells (13.1% of the cells with > 10 species) as possible areas for shark conservation (Fig. 5). Those priority areas were found in the Mediterranean Sea, North Sea, the coast of Australia, Brazil, Chile, European countries, Indonesia, Japan, Libya, Morocco, Mozambique, New Zealand, Peru, South Africa, Sri Lanka, Taiwan, Turkey, and the United States.

### Correlation between the community diversity indices

**Figure 6:**
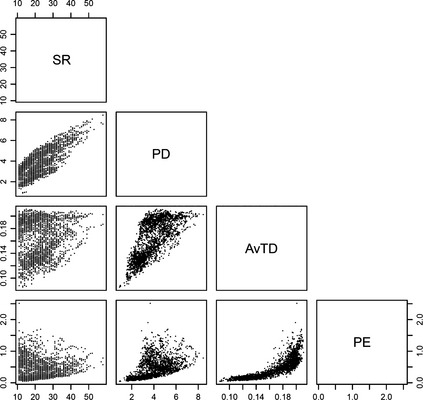
Scatterplots of relationships among species richness (SR), Faith's phylogenetic diversity (PD), average taxonomic distinctiveness (AvTD) and phylogenetic‐clade evenness (PE). Each scatterplot was compared with the designated index corresponding to the row and column. The color of each dot represents the degree of latitude of each cell.

We examined the relationships among PD, AvTD, PE and species richness using the 5048 cells in the shark study. Species richness was highly correlated with PD (*r* = 0.706), but was not correlated with the indices of species composition: correlation with AvTD and PE were 0.121 and −0.033, respectively (Fig. 6). PD and AvTD were highly correlated with each other (*r* = 0.746), how there was no clear linear relationship. Communities that had large PD values also had large AvTD values, whereas communities with large AvTD values had a wide range of PD values. PE was correlated with PD (*r* = 0.559) and even more so with AvTD (*r* = 0.831). Most communities with large PE values had large AvTD values, whereas communities with large AvTD values had a wide range of PE values. In the other extreme, communities with low AvTD values had low PE values and large PS values, the inverse of PE. Communities with large PS values had a wide range of AvTD values. This implies a high resolution of PS in identifying phylogenetically skewed communities.

## Discussion

Our study included 236 shark species in a single analysis on a global scale, which is the most inclusive study to date. Shark species are found almost everywhere in the world, with a diverse array of habits and habitats and thus their conservation must acknowledge their life history strategies. Phylogenetically informed conservation priorities have been widely suggested for many species, on local scales. Several studies have successfully identified shark phylogenetic structure on a local scale. Information is lacking, however, on the global patterns of phylogenetic diversity in sharks and many other marine species. We identified the global patterns of phylogenetic diversity and determined phylogenetic diversity hotspots for sharks. We can now suggest priorities for phylogenetic conservation for sharks, by combining the hotspots of phylogenetic diversity with information on species richness. By including nearly half of all shark species diversity in a single analysis, our main goal was to examine the global patterns of shark community diversities. Furthermore, we aimed to provide information regarding species distributions that may be useful for biologists establishing international conservation strategies across all sharks.

Almost all animal groups are more diverse in tropical environments. Greater species diversity is due to the greater evolutionary time in the tropics, probably caused by shorter generation times, faster mutation rates, and faster selection at greater temperatures (Rohde 1992). However, for sharks, both species richness and phylogenetic diversity were highest on continental shelves and at intermediate latitudes. Phylogenetic diversity, taxonomic distinctiveness and clade evenness were lower in the tropics, due to phylogenetic bias toward specific families in tropical communities.

There are some caveats for this study. First, in the Global Shark Distribution Database, sampling effort and distribution information is not equal across the globe (Lucifora et al. 2009). A species may be absent in certain waters simply because the area is hard to reach or borders a politically unstable region. Thus, the global pattern of species richness may be distorted by the heterogeneous survey effort. The other indices of community diversity may be less sensitive to the heterogeneous survey effort, because they measure the phylogenetic structure of species compositions, rather than the numbers of species. Vélez‐Zuazo and Agnarsson (2011) built the phylogeny of sharks, including 229 species using five genes, as a tool for comparative biological analyses of sharks. To include as many taxa as possible, we built the phylogeny using the most well‐studied sequence data, COI. COI is a mitochondrial marker with fast substitution rates, which gives the best resolution on the branches of a tree. However, COI sequence differences are too small to be detected between closely related species. Using only COI may bias the phylogenetic distinctness metrics. To avoid stochasticity, most phylogenetic trees are based on several molecular markers. Our results would be much stronger if we can build multiple phylogenetic trees using different molecular markers, which revealed similar phylogenetic patterns.

Figure 4 shows that the outer margins of continental shelves have higher PD, AvTD, and PE of shark than the near‐coastal areas. It may be because that the outer margin of continental shelve is an ecotone between neritic and deep sea faunas. Besides, it may be also due to the high species richness in the outer margins of continental shelves (Fig. S1C). The pattern of higher value in outer margins of continental shelves was especially observed for PD, which is mathematically related to species richness. In northwestern Africa, one of the high shark PD areas in the world, the highest PD was observed off the coast of Gambia. Although Gambia has a tiny shoreline, the estuary of Gambia River, a major river in western Africa, provide sufficient nutrients to the marine species, which may cause the high species richness and PD off the coast of Gambia.

**Figure 7:**
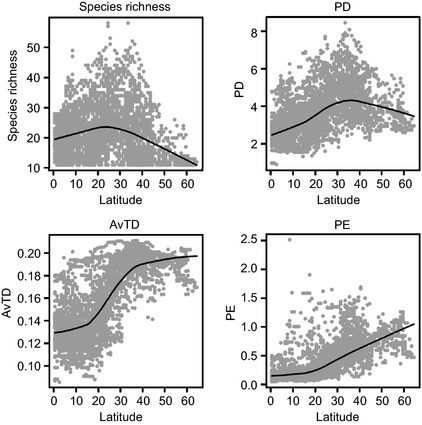
Scatterplots of relationships between latitude and species richness, Faith's phylogenetic diversity (PD), average taxonomic distinctiveness (AvTD), and phylogenetic‐clade evenness (PE). The line in each scatterplot represents locally weighted scatterplot smoothing (LOWESS) curves.

The global pattern of phylogenetic diversity in species compositions was different from the pattern of species richness. Species richness of sharks peaked in intermediate latitudes between 20 and 40° and became very low in high latitude areas, which was similar to other pelagic taxa (Lucifora et al. 2011). We conducted an in‐depth analysis between the metrics and latitude to clearly show different patterns of distribution of the metrics. Species richness was negatively correlated with latitude, whereas PD, AvTD, and PE were positively correlated with latitude (Fig. 7). Positive trends were observed for all indices in low latitude areas. However, species richness and PD reached a peak at midlatitude areas and began to decrease at 20–30 and 30–40°, respectively. In contrast, AvTD and PE showed an opposite trend, they began to increase sharply from midlatitude areas. AvTD was constant from 30°, although PE maintained an increasing trend in high latitude areas. The lowest values of PD, AvTD, and PE were located in low latitude areas. As for species richness, the distribution of low values covered a wide range, from high to low latitudes. The highest values of the proposed index, PE, were distributed widely from 10 to 40° latitude, which was wider than PD and AvTD (ranged between 30 and 40°). In the tropical zone, consistently low values of PE were observed, but this was not the case for AvTD. This result implies that although sharks are all skewed to a few specific clades in tropical regions, taxonomically distinctive species can be found in other areas. In contrast, there was a more diverse distribution of PE than AvTD in temperate zones.

To contemplate the practical consequences of observed patterns of distribution of the metrics on shark conservation strategies, we compared our findings with the distribution of threatened species presented by Dulvy et al. (2014). They conducted the systematic analysis of threat for a globally distributed lineage of sharks, rays, and chimeras, and shown the distribution of threatened species (Figure 9 of Dulvy et al. 2014). They revealed the magnitude of threat in mainly the pelagic ocean and coastal areas, the Indo‐Pacific Biodiversity Triangle, the Mediterranean Sea and the Red Sea. Our conservation priority areas were found mainly in the coastal regions, including the islands of Indonesia and the Mediterranean Sea. The coastal areas have the highest species richness and are ecotones between neritic and deep sea faunas, which results in high phylogenetic diversity. Besides protecting the hotspots of threatened species, it is necessary to take full account of phylogenetic patterns of species composition for shark conservation.

We found phylogenetic diversity of sharks was high in high latitude areas. Although PD, which is closely related with species richness, also peaked at latitudes between 20 and 40°, it did not decrease sharply in high latitude (up to 60°N in the analysis) areas like species richness did. However, both AvTD and PE were found to be very high in high latitude areas. Our findings suggest that when establishing conservation activities, it is important to take full account of phylogenetic patterns of species composition and not solely use species richness as a target. Protecting areas of high phylogenetic diversity in sharks, which were identified in this study, could form a broader strategy for protecting other threatened marine species.

## Conflict of Interest

None declared.

## Supporting information


**Figure S1.** Global patterns of shark species richness.

**Figure S2.** Top 5% of (A) phylogenetic diversity (PD), (B) average taxonomic distinctiveness (AvTD), (C) phylogenetic‐clade evenness (PE), and (D) species richness used in our analysis (mitochondrial DNA COI sequences available species).

**Data S1.** Mitochondrial DNA COI sequences used to conduct the shark phylogenetic reconstruction.
