# Supplementary figures and images for: Global pattern of phylogenetic species composition of shark and its conservation priority
Source: Ecol Evol. 2015 Sep 23;5(19):4455–65. doi: 10.1002/ece3.1724 (PMC4667821; doi:10.1002/ece3.1724)

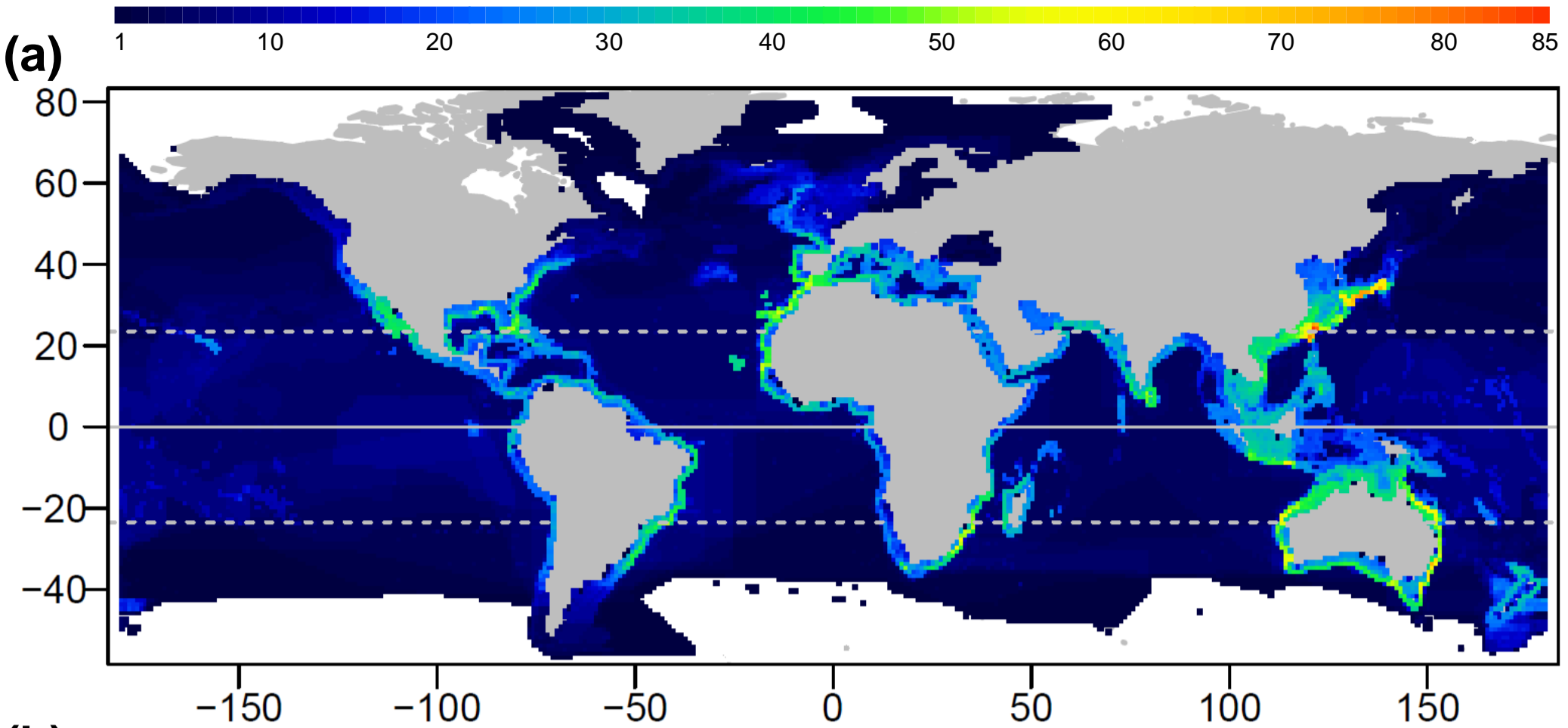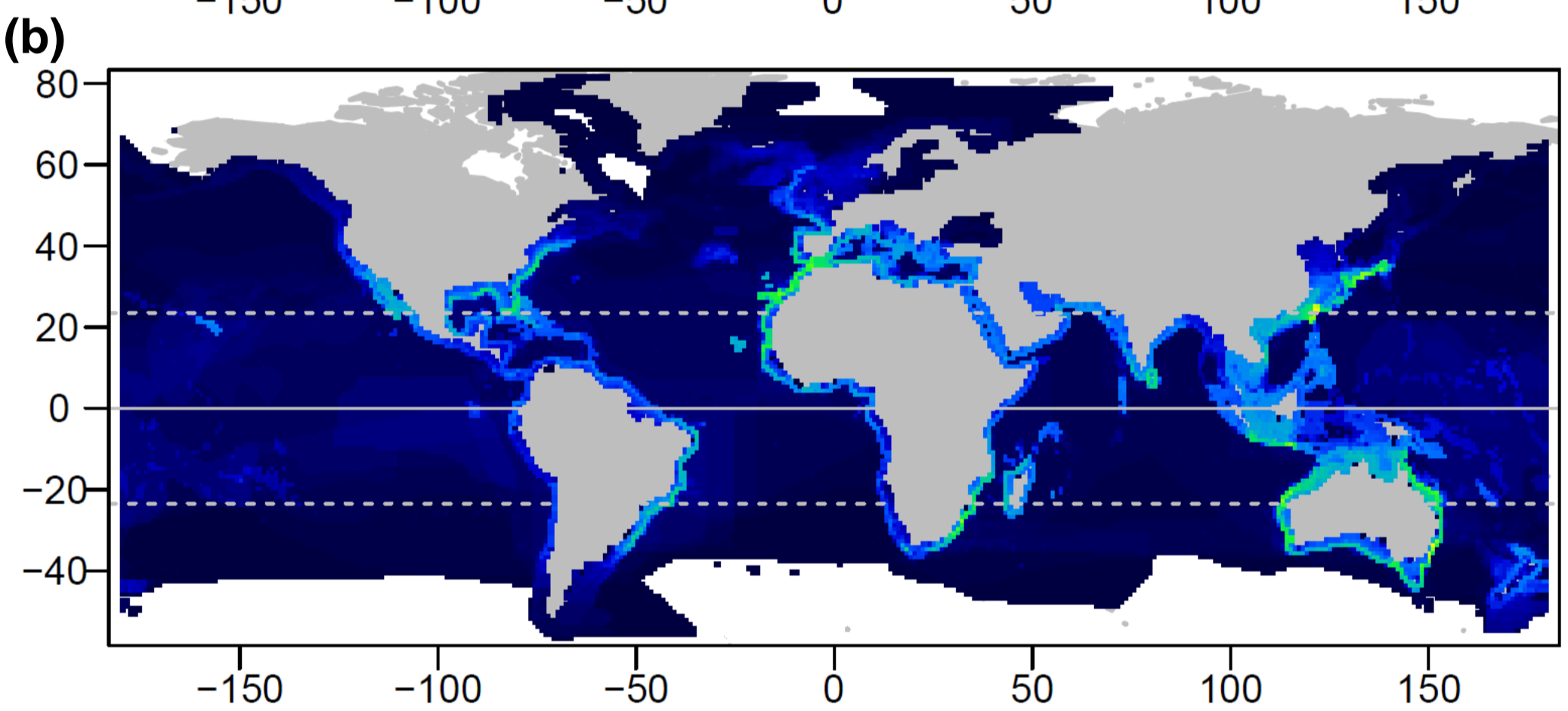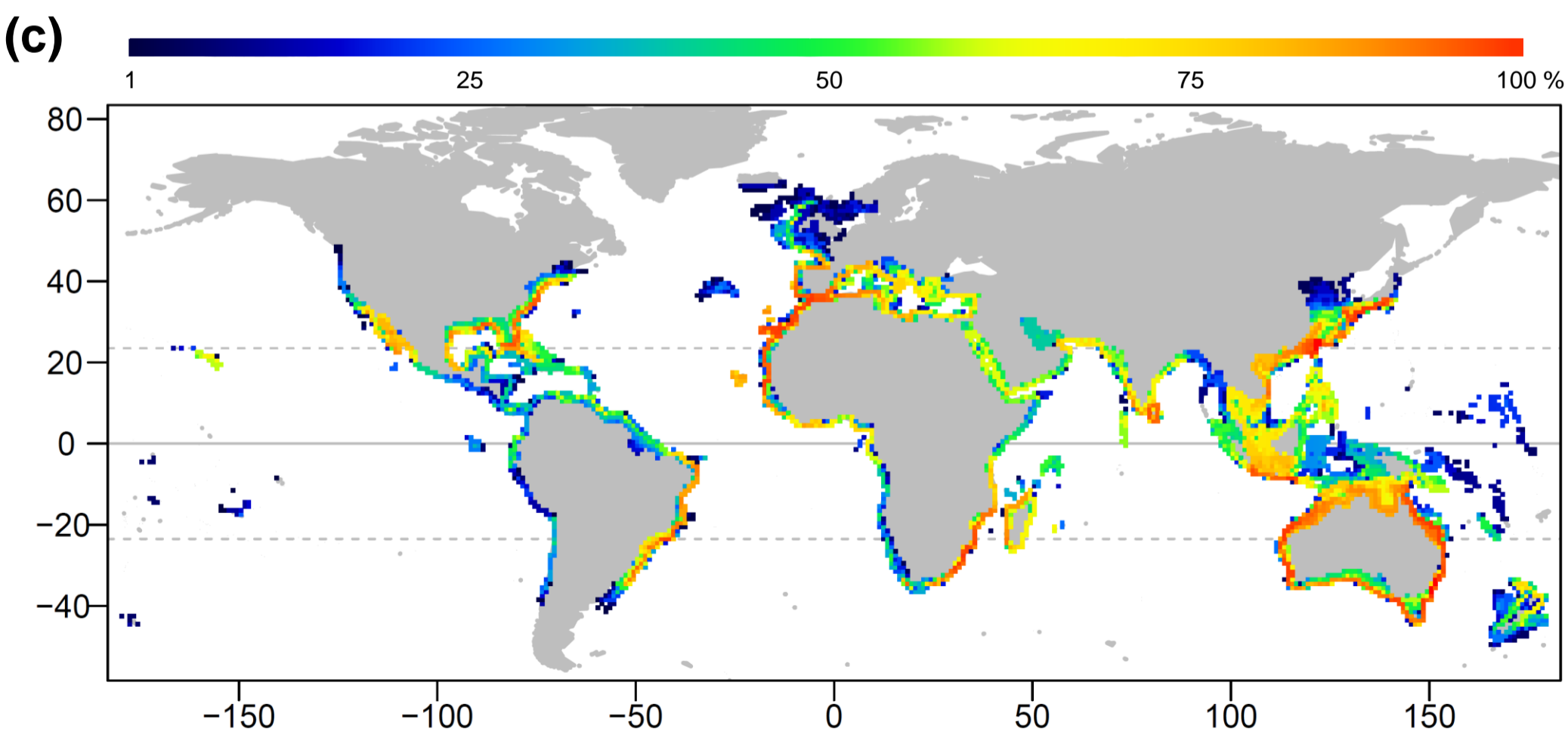

Supplement: Supplementary file 1 — Figure S1. Global patterns of shark species richness. [file ECE3-5-4455-s001.pdf]

**(a)**

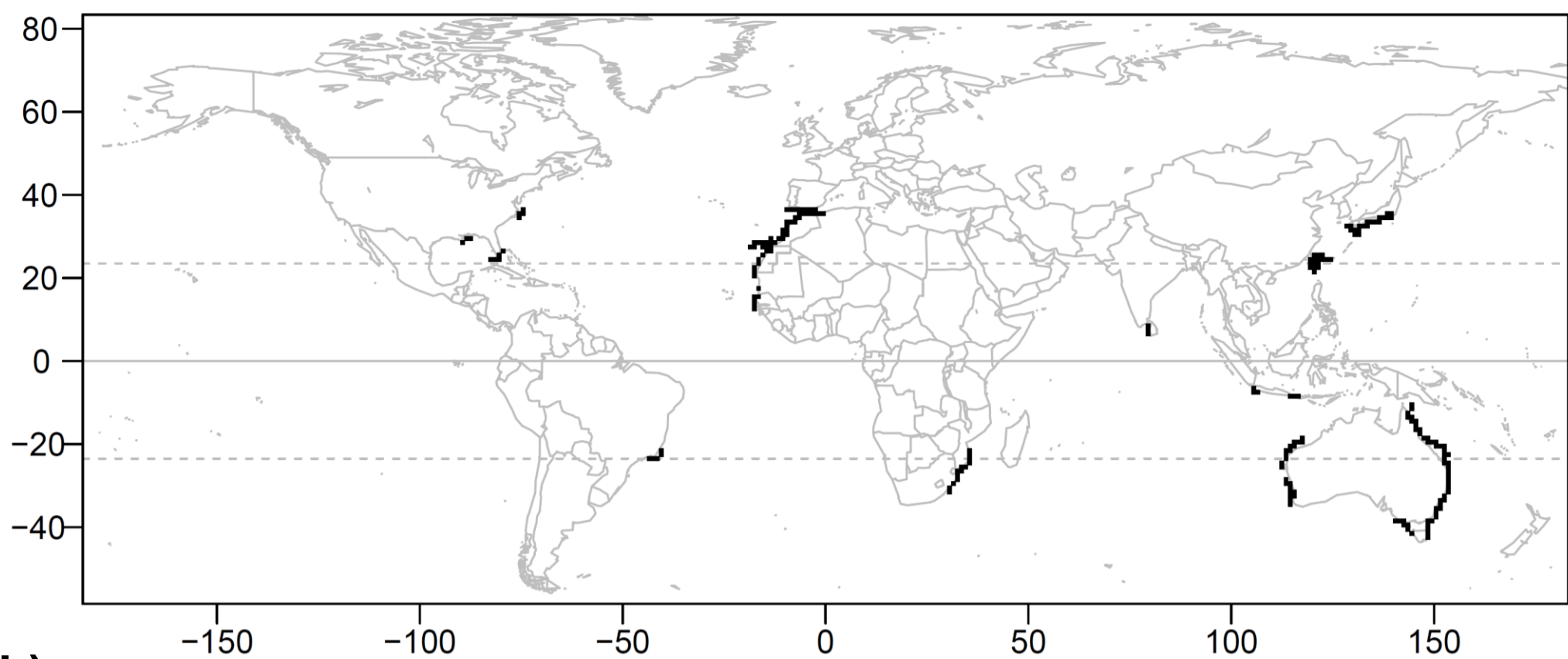

**(b)**

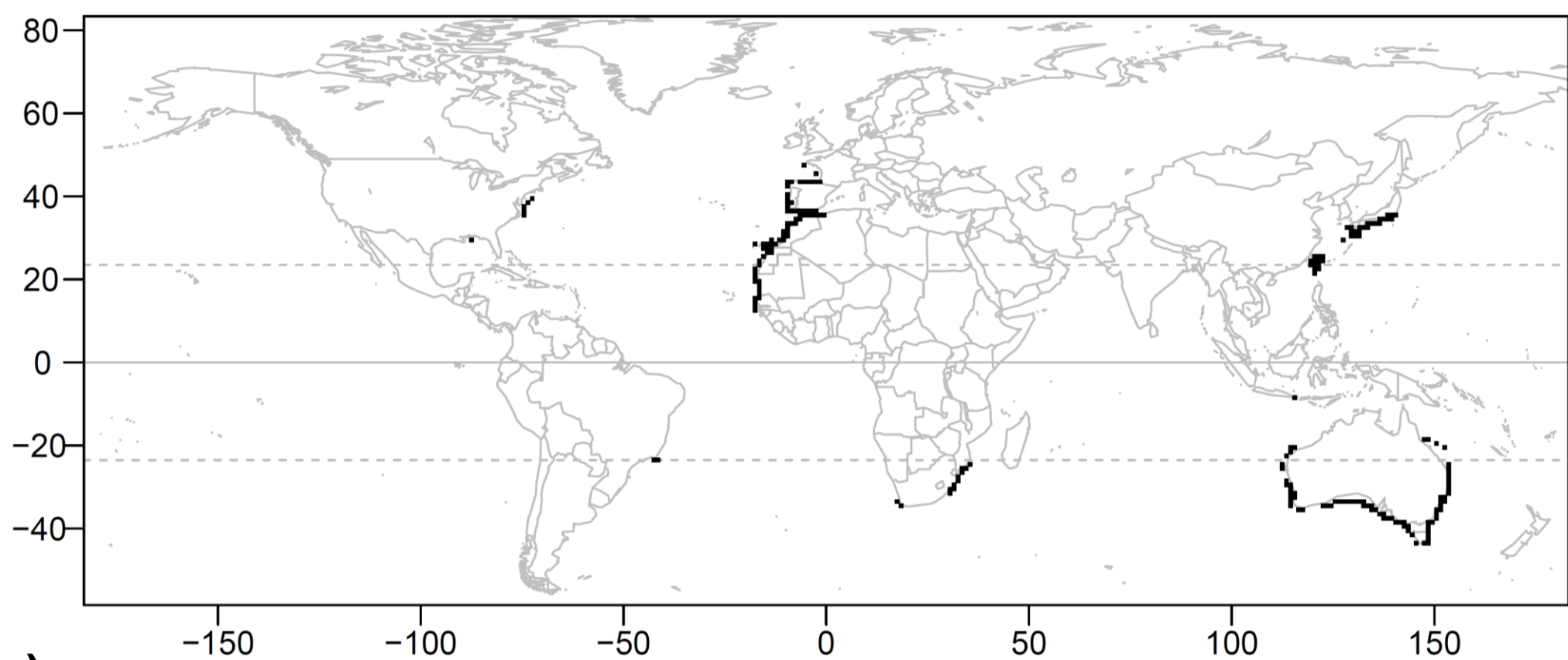

**(c)**

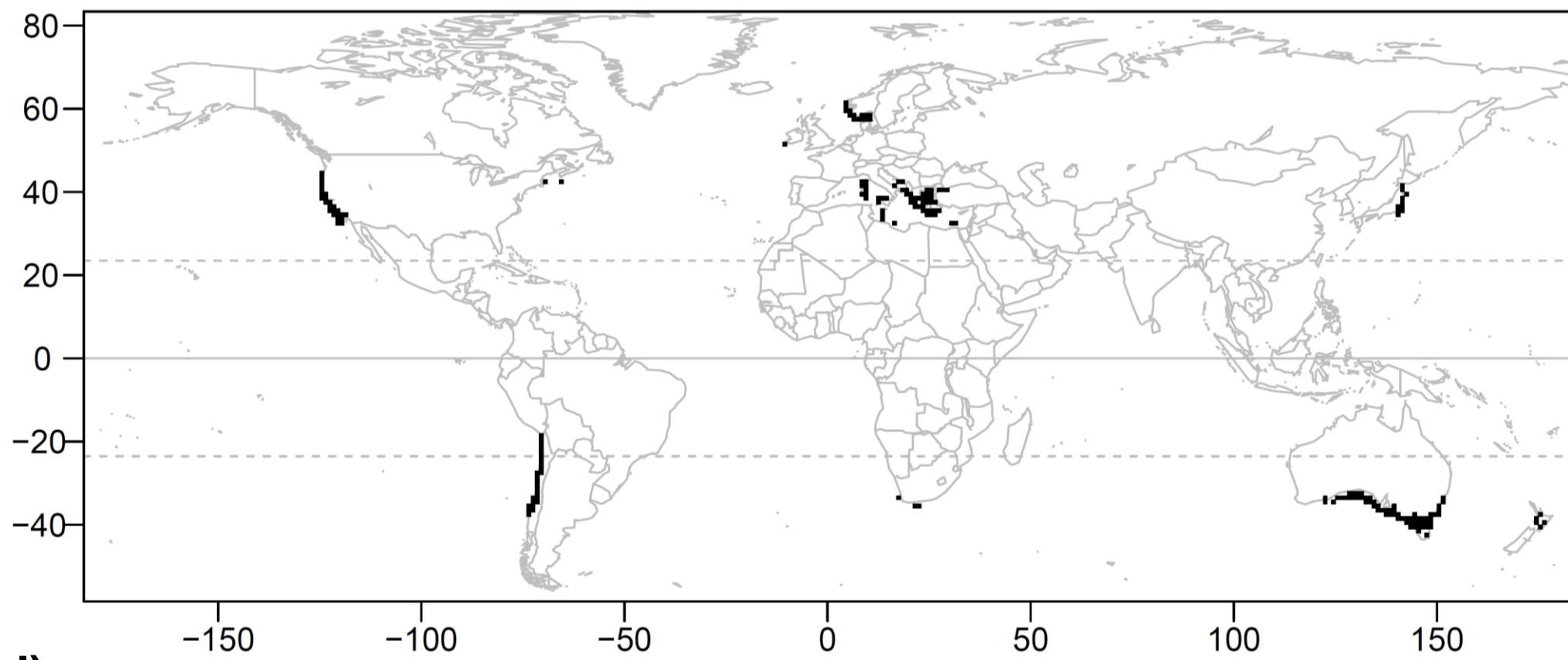

**(d)**

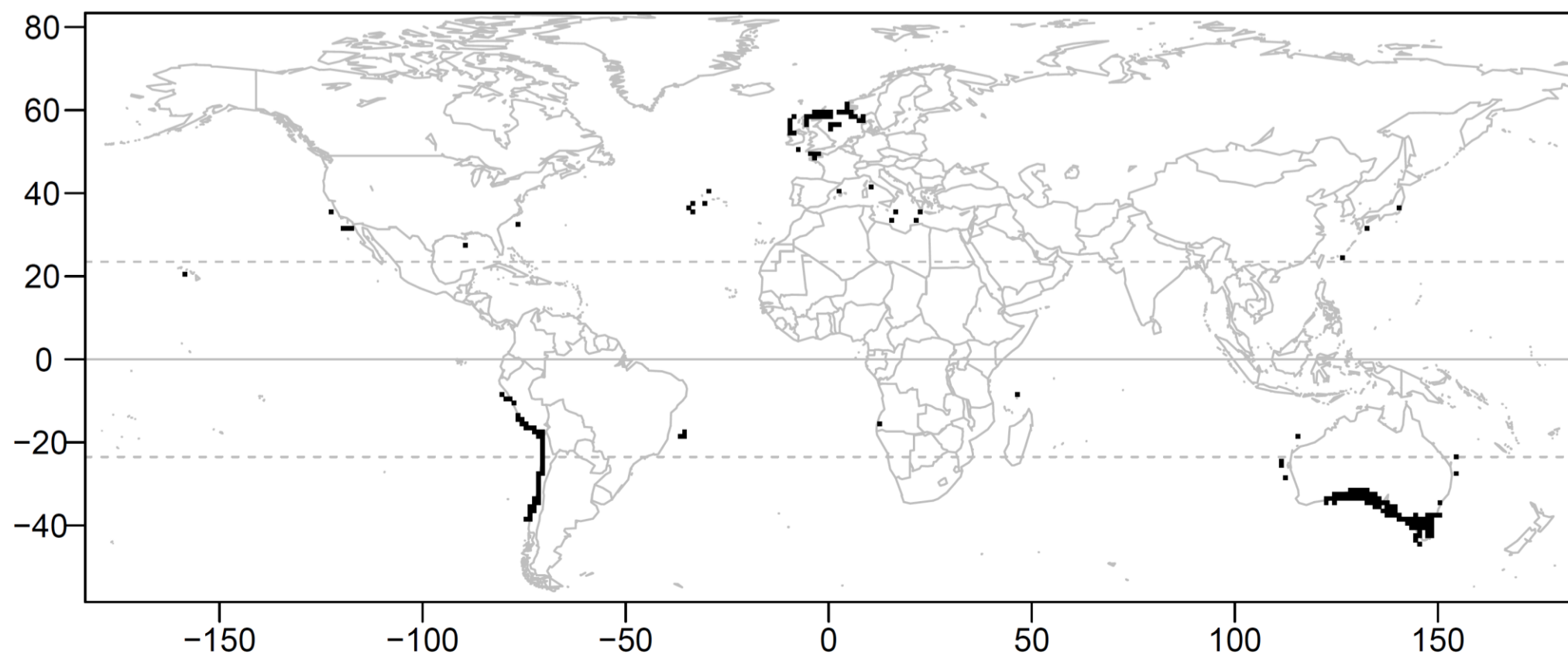

Supplement: Supplementary file 2 — Figure S2. Top 5% of (A) phylogenetic diversity (PD), (B) average taxonomic distinctiveness (AvTD), (C) phylogenetic‐clade evenness (PE), and (D) species richness used in our analysis (mitochondrial DNA COI sequences available species). [file ECE3-5-4455-s002.pdf]
